# Critical assessment of influenza VLP production in Sf9 and HEK293 expression systems

**DOI:** 10.1186/s12896-015-0152-x

**Published:** 2015-05-16

**Authors:** Christine M Thompson, Emma Petiot, Alaka Mullick, Marc G Aucoin, Olivier Henry, Amine A Kamen

**Affiliations:** National Research Council Canada, Human Health Therapeutics, Montréal, Canada; Ecole Polytechnique de Montréal, Montréal, Canada; Laboratoire Virologie et pathologies Humaine (VirPath), EA4610 Lyon, France; University of Waterloo, Waterloo, Canada; Department of Bioengineering, McGill University, 817 Sherbrooke St. W. Macdonald Engineering Building, Room 387, Montréal, Canada

**Keywords:** Influenza vaccines, Virus like particles (VLPs), Insect cells, Mammalian cells, Process development, VLP characterization

## Abstract

**Background:**

Each year, influenza is responsible for hundreds of thousand cases of illness and deaths worldwide. Due to the virus’ fast mutation rate, the World Health Organization (WHO) is constantly on alert to rapidly respond to emerging pandemic strains. Although anti-viral therapies exist, the most proficient way to stop the spread of disease is through vaccination. The majority of influenza vaccines on the market are produced in embryonic hen’s eggs and are composed of purified viral antigens from inactivated whole virus. This manufacturing system, however, is limited in its production capacity. Cell culture produced vaccines have been proposed for their potential to overcome the problems associated with egg-based production. Virus-like particles (VLPs) of influenza virus are promising candidate vaccines under consideration by both academic and industry researchers.

**Methods:**

In this study, VLPs were produced in HEK293 suspension cells using the Bacmam transduction system and Sf9 cells using the baculovirus infection system. The proposed systems were assessed for their ability to produce influenza VLPs composed of Hemagglutinin (HA), Neuraminidase (NA) and Matrix Protein (M1) and compared through the lens of bioprocessing by highlighting baseline production yields and bioactivity. VLPs from both systems were characterized using available influenza quantification techniques, such as single radial immunodiffusion assay (SRID), HA assay, western blot and negative staining transmission electron microscopy (NSTEM) to quantify total particles.

**Results:**

For the HEK293 production system, VLPs were found to be associated with the cell pellet in addition to those released in the supernatant. Sf9 cells produced 35 times more VLPs than HEK293 cells. Sf9-VLPs had higher total HA activity and were generally more homogeneous in morphology and size. However, Sf9 VLP samples contained 20 times more baculovirus than VLPs, whereas 293 VLPs were produced along with vesicles.

**Conclusions:**

This study highlights key production hurdles that must be overcome in both expression platforms, namely the presence of contaminants and the ensuing quantification challenges, and brings up the question of what truly constitutes an influenza VLP candidate vaccine.

**Electronic supplementary material:**

The online version of this article (doi:10.1186/s12896-015-0152-x) contains supplementary material, which is available to authorized users.

## Background

As reported by the World Health Organization, seasonal influenza is responsible for approximately 500 million cases of infection and between 250,000 to 500,000 deaths each year [1]. Currently, vaccination remains the most proficient strategy to prevent infection and to battle the persistent threat of influenza epidemics. Egg-based production has remained the standard method to produce seasonal influenza vaccines since the 1950s; however, the serious threat of an outbreak of pandemic avian flu and the influenza H1N1 pandemic of 2009 have highlighted the limitations associated with this manufacturing method [2,3]. These drawbacks include the dependence on the availability of eggs for production and their associated limitations with regards to scalability (approximately 1 vaccine dose per egg), the relatively long 6-month period from strain isolation to final dose formulation and validation [4], and the difficulties with growing isolated avian strains. Cell culture based production has been proposed to overcome such limitations; these processes are easier to scale-up and, through the standardization of production methods, allow for increased vaccine production. Moreover, cell culture processes have the potential to reduce manufacturing time by several weeks with a faster start-up time [5].

Seasonal influenza vaccines derived from cell culture are gaining increased attention. In November 2012, the first seasonal vaccine produced in cell culture (Flucelvax, Novartis) was approved by the FDA. In January 2013, the first trivalent influenza vaccine, Flublok (Protein Science Corporation) made in insect cells using a recombinant baculovirus expression system, was also approved. These recent advances reflect an important trend of adopting modern cell culture manufacturing in the influenza vaccine industry. It is highly supported by public health and regulatory agencies that promote strategies to improve responses to emerging infectious diseases.

One of the most promising approaches is the use of recombinant-based virus-like particle (VLP) vaccines [6,7]. Influenza VLPs are non-infectious and non-replicating empty viral particles lacking viral genetic material, which display intact and biochemically active antigens on their surface. They can be composed of one, or different combinations, of the viral antigens and structural influenza proteins: HA, NA, M1 and M2 [6,8-11].

Immunizations with influenza VLPs to protect against either seasonal or pandemic influenza strains have shown promising results [8,12-14]. VLPs have a stronger safety profile than native infectious viruses produced either in egg-based or cell-based systems. No infectious virus potentially harmful to humans is used as a seed inoculant or produced, thus production processes do not require the use of high level containment facilities. With more clinical trials underway for VLP vaccines [15,16], there is a critical need for the scientific and engineering communities to tackle the specific challenges associated with the bioprocessing aspects of VLP production.

Thus far, different production processes have been studied for influenza VLPs. They vary according to the viral strain produced, the type of gene delivery system used and the host-cell expression system. Influenza VLPs have been produced in mammalian, insect and plant cell platforms using a variety of vectors and gene delivery techniques [17-19]. The majority of VLP productions have been done in insect cells with the baculovirus expression vector system (BEVS) [8,18,20,21]. However, this system suffers from contamination with baculovirus (BV) particles [14,22] in final vaccine preparations. This contamination has been known for a long time and can represent up to 5% of the total proteins in final purified VLP preparations [14] and 4.5 × 10^6^ BEVS per vaccine dose [22]. Baculovirus are non-replicative in mammalian cells, but they have been reported to trigger an innate immune response. Although there is some evidence that baculovirus is safe for immunization [22,23], in terms of process development and evaluation of the immunization efficiency from VLPs alone, such high level of contaminant presents a problem. Alternative methods of VLP production in mammalian cells, using either plasmid transfection or baculovirus gene transfer in mammalian cells (BacMam) have thus been developed. The second problem posed by the insect cell platforms is product glycosylation, as insect cells do not allow proper sialylation of the proteins [24]. Different groups are now working on the introduction of such glycosylation pathways in insect cell systems.

Most studies thus far have focused on the proof of concept of VLP production and the evaluation of influenza VLPs immunogenicity to support their candidacy as potential vaccines [8,12] and as a model to gain insight into the requirements for influenza virus budding [25]. Nevertheless, to date, limited attention has been paid to the specific challenges posed by the manufacturing of VLPs. However, studies of this nature are starting to surface [18,19,22,26].

In line with the theme of studying the bioprocessing of influenza VLPs, this study assessed two platforms for VLP production, the insect (Sf9) and mammalian (HEK293) suspension and serum-free production platforms with influenza gene transfer by baculovirus infection and transduction, respectively. VLP production and contaminant levels (VLP/mL, BV/mL, μg HA/mL, HA units/mL), as well as the challenges associated to each production systems are discussed.

## Methods

### Cells and medium

The HEK293 cell line used for VLP production was previously adapted to suspension and serum-free culture [27]. Working cell banks were made from a vial of the master cell bank, which was developed under Good Manufacturing Practices (GMP). HEK293 cells used for VLP production were grown in shake flasks at 37°C and 5% CO_2_, in animal component and serum free SFM4Transfx 293™ (HyQ) medium (HyClone, Waltham, MA, USA). Insect *Spodoptera frugiperda* (Sf9) cells were maintained in serum free Sf900 II medium (GIBCO, Burlington, ON, Canada), in shake flasks at 27°C with an agitation rate set at 110 rpm. Cell density was monitored using the Cedex Cell Counter (Innovatis Roche Applied Science, Penzberg, Germany).

### Construct design of the gene transfer system

#### Baculovirus BacMam for mammalian cell production system (HEK293)

The recombinant baculovirus used for HEK293 cell transduction (referred as BacMam PR8) was previously described in Tang et al. [19] and kindly donated by Dr. Ted Ross (University of Pittsburgh). One recombinant baculovirus was used to drive the expression of HA, NA and M1 genes from H1N1 A/Puerto Rico/8/1934 influenza strain under the control of individual CMV promoter. BacMam PR8 also contained a Green fluorescent protein (GFP) reporter gene under the CMV promoter and a VSV-G protein under polyhedron promoter control in order to improve cell transduction. A working stock of BacMam PR8 baculovirus was produced by generation of P0 BacMam PR8 stock in Sf9 cells with bacmid transfection and two subsequent BacMam PR8 amplifications in Sf9 cells (the VP/mL and IVP/mL of each stock can be found in the Additional file 1: Table S1).

#### Baculovirus construction for insect cells production system (Sf9 cells)

For production in insect cells, co-infection with three baculoviruses each carrying influenza proteins, HA, NA or M1 was chosen. The influenza proteins were under polyhedron promoter (polh) control for expression in insect cells. The construction of vectors for further generation of P0 baculovirus stocks through Sf9 cells transfection was performed as follows: the DNA sequence of H1N1 A/Puerto Rico/8/1934 HA (AB671289.1), NA (AB671290.1) and M1 (CY033578.1) were obtained from NCBI’s influenza database. Influenza gene sequences were inserted between XbaI and BglII enzyme restrictions sites and further in a pUC plasmid by BioBasic (Markham, Canada). Each influenza gene, flanked by XbaI and BglII sites, was further inserted in the pVL1393 plasmid, belonging to the commercial BaculoGold™ system (BD bioscience, Franklin Lakes, USA) used for the construction of baculovirus allowing expression of recombinant proteins in Sf9 cells. Each of the three plasmids, respectively named pVL1393-HA, pVL1393-NA and pVL1393-M1, were co-transfected with baculovirus DNA to produce recombinant baculoviruses referred to as Bac-HA, Bac-NA, and Bac-M1. Similarly, to produce the BacMam PR8 virus stock, each Bac-HA, Bac-NA and Bac-M were passaged twice in Sf9 cells to produce a P2 working viral stock used for VLP production (VP/mL and IVP/mL of each stock can be found in the Additional file 1: Table S1).

### VLP production

For both cellular platforms, during the production optimization phases, cultures were sampled once to twice a day and cell density, viability and average cell diameter were measured using the Cedex Cell Counter (Innovatis Roche Applied Science, Penzberg, Germany).

#### Mammalian HEK293 cell platform

For VLP production in HEK293 cells, transduction with BacMam PR8 was performed at a cell density of 1.5-2.0×10^6^ cells/ml. An MOI of 60 was used (MOI calculations were based on infectious baculovirus titers). Production runs were carried out in shake flasks with working volumes of 60–400 ml. In production runs, butyric acid was added at a final concentration of 5 mM at the time of infection. After 48 hours post-infection, culture broth samples were clarified by slow centrifugation at 300 × g for 5–10 minutes. Culture supernatant samples were concentrated via sucrose cushion ultracentrifugation and analysed by Western blot, HA assay, Negative Stain Transmission Electron Microscopy (NSTEM) and Single Radial Immuno-diffusion assay (SRID).

#### Insect Sf9 cell platform

VLP productions in Sf9 cells were performed in shake flasks with working volumes of 60–400 ml. Cells were infected with P2 working stocks at a density of 2.0-2.5 × 10^6^ cells/ml with a total MOI of 0.3-2.1 at 27°C (MOI calculations were based on infectious baculovirus titers). Cells were harvested after 48 hours post-infection, when the viability started to decline below 70-50%. Upon harvest, the supernatant samples were collected with slow centrifugation at 300 × g for 5–10 minutes, then concentrated via sucrose cushion ultracentrifugation and analysed by Western blot, HA assay, NSTEM and SRID.

### VLPs extraction from cell pellet

The cell pellets obtained after slow centrifugation were re-suspended in a solution of PBS and 10 μg/ml of TPCK-trypsin and slowly agitated at 37°C for 30 minutes to produce cell pellet wash. Cells were pelleted via slow centrifugation and the supernatant was collected for analysis by Western blot, HA assay and NSTEM. A cell lysis extraction was also performed after this first treatment by one freeze-thaw cycle. The cell lysis supernatant was collected by centrifugation at 1000 × g for 5 min and then analysed using Western blot, HA assay and NSTEM.

### Influenza virus production

Influenza H1N1 A/PR/8/1934 virus was produced in house as described in Petiot et al. [28] and used as standard for the Western blot, HA assay and NSTEM images.

### VLP concentration and purification

#### Sucrose cushion ultracentrifugation

The VLP supernatants were sublayered with 10 ml of a 25% sucrose solution prepared in 20 mM Tris–HCl (pH 7.5). The maximal VLP volume loaded was of 200 ml of culture supernatants, centrifuged at 4°C and 37 000 × g for 3 hours (Sorvall Discovery SE 100 ultracentrifuge, A621 rotor, Thermo Scientific, Waltham, USA). The ultracentrifugation supernatants were discarded and the VLPs were collected as pellets, re-suspended in 20 mM Tris–HCl, 1% sucrose and 2 mM MgCl_2_. Ultracentrifugation purified samples were stored overnight at 4°C, then aliquoted and stored either at −80°C or 4°C. This purification step allowed to concentrate VLP production samples up to 20-75×.

#### Iodixanol gradient ultracentrifugation

Gradient iodixanol purification was done from two working stock solutions of iodixanol, one with 40% w/v and one with 25.5% w/v. These solutions were prepared from Optiprep iodixanol solution (Axis-Sheild, Oslo, Norway) with 60 mM Tris–HCl (pH 7.5). VLP concentrated solutions from the sucrose cushion purification step were combined to the 25.5% iodixanol working stock and subjected to slow mixing to give a final iodixanol concentration of 20% and a VLP dilution of 4.7×. The VLP-iodixanol solution was then loaded into 13 ml ultracentrifuge tubes, sealed and centrifuged at 350 000 × g for 6 hours at 4°C (Sorvall Discovery SE 100 ultracentrifuge, 65 V13 rotor, Thermo Scientific, Waltham, USA). After ultracentrifugation, 1 ml fractions were taken from the bottom of the tubes and weighed to determine the density and iodixanol concentration. Each fraction was then analysed by Western blot, HA assay and NTSEM.

### VLP quantification and detection

#### Western blot

Samples were loaded on a Bio-Rad mini-protean Tris-Glycine 4-15% gel with 1× running buffer (25 mM Tris, 192 mM Glycine, 3.5 mM SDS). The gel was run for 40 minutes at 200 V and then washed with Towbin transfer buffer (25 mM Tris, 192 mM Glycine, 20% Methanol pH 8.3). Semi-dry protein transfer to nitrocellulose membrane was performed using a Bio-Rad Trans-Blot 3D semi-dry transfer cell (Hercules, CA, USA) for 1 hour at 10 V. Membranes were then blocked for 1 hour in 5% milk solution (1× TBS, pH 7.5: 50 mM Tris, 150 mM NaCl, 0.1% Tween-20, 5% milk). The membrane was washed with 0.1% Tween TBS at pH 7.5 and then incubated overnight at 4°C with its respective antibody (conc. 1/1000). The secondary antibody incubation (conc. 1/1000) lasted 1 hour at 4°C. Primary antibodies used for HA, NA, M1 and GP64 were as follows: anti-HA and anti-NA, polyclonal sheep serum (ref. 03–242 and 04–230, respectively, NIBSC, London, UK); anti-HA monoclonal mouse (ref. sc-80550, Santa Cruz Biotechnology, USA); anti-M1 monoclonal mouse antibody (ref. ab22396, Abcam, Cambridge, USA); anti-GP64 monoclonal mouse antibody (ref. 14-6995-81, eBioscience, San Diego, USA). The secondary antibodies used were Donkey anti-sheep and Donkey anti-mouse (ref. 713-005-003, 715-005-150, respectively) IgG HRP from Jackson ImmunoResearch (Westgrove, USA). Images were acquired with a DS Image Station 440CF (Kodak, Rochester, USA).

#### Hemagglutination assay

The HA assay was completed in 96 well v-bottom plates. Wells were filled with 100 μl of 1× PBS solution in rows A-H, columns 2–12. Next, 29.3 μl of 1× PBS was loaded into wells B1, B2, D1, D2, F1, F2, H1 and H2. Each sample took up two rows (A and B, C and D, etc.) and was serially diluted with VLP samples from the supernatant, sucrose cushion and iodixanol purifications. 100 μl of VLP sample was loaded into wells 1 and 2 of rows A, C, E, and G, then 70.7 μl into wells 1 and 2 of rows B, D, F and H. The VLP sample was then serially diluted and 100 ul of 5-day old chicken red blood cells at a concentration 2×10^7^ cell/ml were added to each well in the plate. The plates were left in a covered plastic container for 3 hours to overnight at room temperature and scored. The amount of HA in units HA/ml was calculated with the following correlation:

$$ \frac{Log\ HA\  units}{100\ ul}= \log \left( dilution\  factor\  at\  last\  well\  agglutinated\right) $$

#### Single radial immunodiffusion assay

A 1% agarose gel solution was prepared and equilibrated at 50°C in a water bath. Agarose gel solution was added with anti-HA sheep serum (ref. 03–242, NIBSC, London, United Kingdom) with a final concentration of 1/1000. The agarose-antibody mixture was casted and left to cool in a Bio-Rad gel-casting module for at least 15 minutes. In the gel, 4 mm wells were punched before addition of 20 μl of sample in each well. For all the SRID experiments, a calibration curve was determined with standard samples of purified recombinant HA of A/H1N1/Puerto Rico/8/1934 strain from Protein Sciences Corporation (Meriden, USA) at concentrations ranging from 7 to 30 μg/ml. All the samples were subjected to detergent treatment for 15 minutes on a rocker platform (1% Zwittergent final concentration). Sample diffusion in the gel took place for 18–24 hours at room temperature. The gel was dried using Whatman #3 filter paper (Kent, United Kingdom), rinsed with deionized water, and stained with Coomassie Blue R-250 for 15 minutes and de-stained using a 7.5% acetic acid and 10% ethanol solution. Result analyses were performed on gel photograph where the diameter of the reference standards and samples were measured using Image-J software (Pixel-aspect ratio = 1, known distance = 4 mm) allowing further calculation of sample concentration through linear correlation.

#### Negative Stain Transmission Electron Microscopy (NSTEM)

NSTEM analysis were conducted at Institut Armand Frappier (Laval, Canada) adapting a method previously described [29]. For each samples, 2 micrographs were counted with samples pre-diluted to have at least 25 VLPs on the grid. The range of VLPs counted was between 25 and 300 particles. The particle count was quantified using the following equation: VLP/ml = (VLP count/latex bead count) × (latex bead concentration × virus dilution).

## Results and discussion

It is important to note that, for all the analysis performed (NSTEM, Western Blot, HA assay, SRID), the samples were concentrated by a sucrose cushion ultracentrifugation step. This concentration step was necessary as the available quantification methods are not sensitive or specific enough to directly quantify total VLPs and protein composition of non-purified culture supernatants. This allowed us to i) reach detectable levels and ii) and reduce the contamination by free influenza and host cell proteins in the sample. Consequently, most of the assays were performed with at least a 40× concentrated sucrose cushion purified samples and all the values presented in the paper correspond to their relative normalized production levels (per/mL).

### VLP morphology and particle quantification

**Figure 1:**
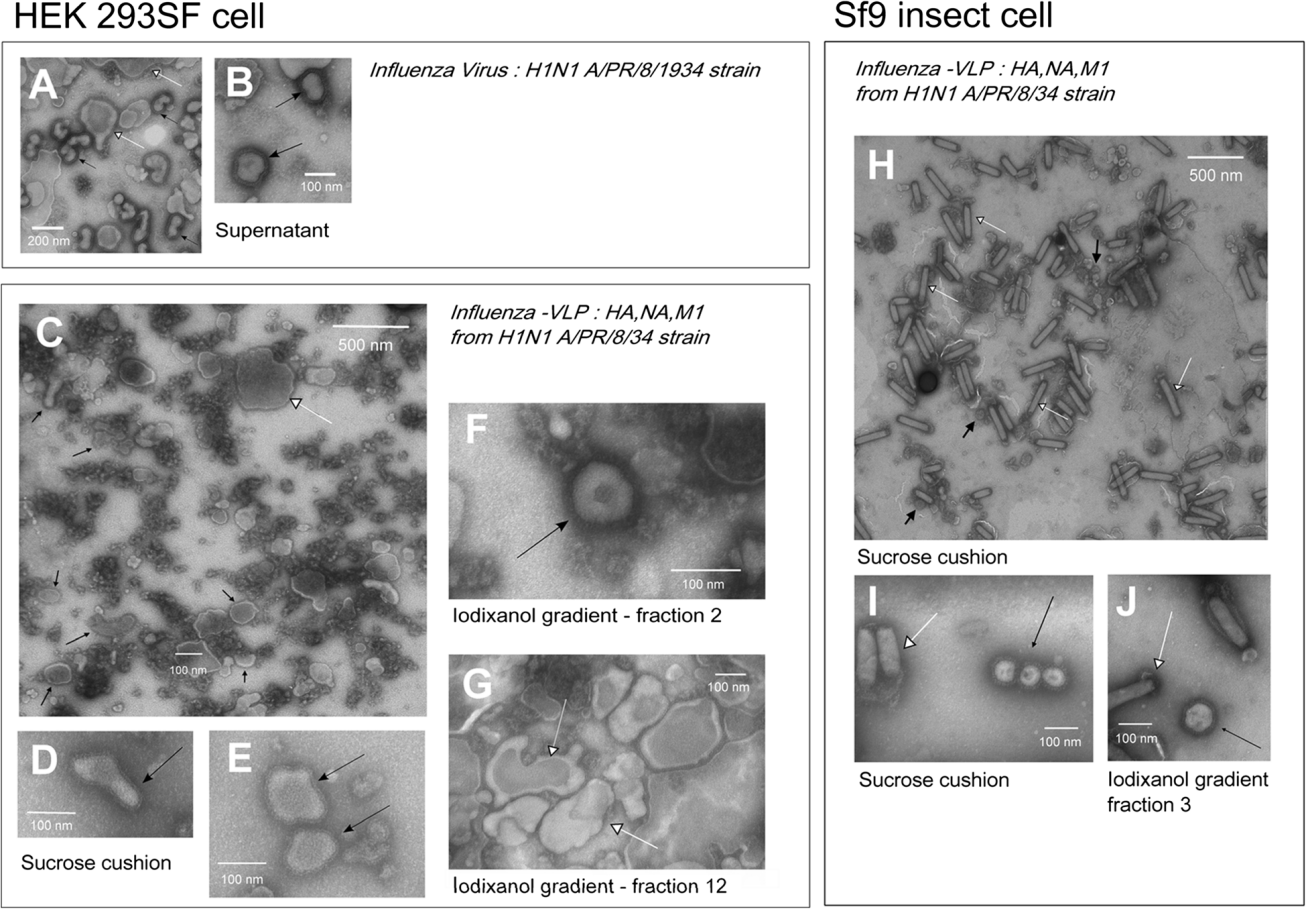
NSTEM images at 40,000× magnification of H1N1 A/Puerto Rico/8/34 **(A** **&** **B)**, H1N1 influenza-VLPs from HEK 293SF cell production **(C**-**G)** or Sf9 cell production **(H**-**J)**. VLPs or influenza virus are pointed by black arrows, baculovirus and cell vesicles are pointed by white arrows. **A** & **B** - Supernatant of H1N1 A/Puerto Rico/8/1934 influenza virus produced in HEK 293SF suspension cells as in Petiot et al. [28]. Cell vesicles carrying influenza glycoproteins could be identified. **C**, **D** & **E** - Sucrose cushion purified samples of HEK 293SF cell production; large view **(C)** and zoom-in **(D** & **E)** images of NSTEM grid. **F** & **G** - Iodixanol purified samples of HEK 293SF cell production. Typical shapes of VLPs and vesicles identified in high density (fraction 12: 1.03 g/ml) and low density (fraction 2:) iodixanol fractions. **H** & **I** - Sucrose cushion purified samples of Sf9 cell production. **J** - Iodixanol purified samples fraction 3, which present the highest number of VLP particles. Baculovirus were co-purified with VLPs in all the iodixanol fractions, even if they were more concentrated in the high density fractions.

Influenza virus and VLP morphology were analysed by visual inspection on NSTEM micrographs, and the concentration of VLP particles were assessed for the comparison of VLP production efficiency in both cellular platforms. Previous studies of influenza virus production in HEK293 cell culture described the virus as pleomorphic particles containing spikes with an average size of 100 +/− 20 nm [30]. Influenza virus consisted mainly of spherical and elongated particles [31]. An example of H1N1 A/Puerto Rico/8/1934 influenza viruses produced in-house on the same HEK293 cell platform [31] is shown in Figure 1. On this basis, VLPs were defined as particles with a fringe of spikes on their membrane, assumed to be influenza glycoproteins HA and NA, with a size range of 100 +/− 50 nm. NSTEM images of VLPs produced with both HEK293 SF and Sf9 cells are shown in Figure 1. Pictures presented are all from purified samples, either sucrose cushion (C, D, E H & I) or sucrose cushion and iodixanol purified (F, G & J).

Figure 1D, E & F present zoomed-in images of VLPs produced with HEK293 cells. The VLPs were similar to influenza virus particles, although their average size ranged from 100 to 500 nm and they displayed the typical morphology of host cell vesicles. VLPs were counted to be at a concentration of 1.5 × 10^8^ VLPs/ml.

Figure 1I & J present zoomed-in images of VLPs produced in Sf9 cells. Their morphology seems to be uniform and close to influenza virus, consisting of spherical particles with an average size close to 100 nm. For these samples, a concentration of 5.85 × 10^9^ VLPs/ml was quantified for Sf9 VLP production.

### VLP composition

**Figure 2:**
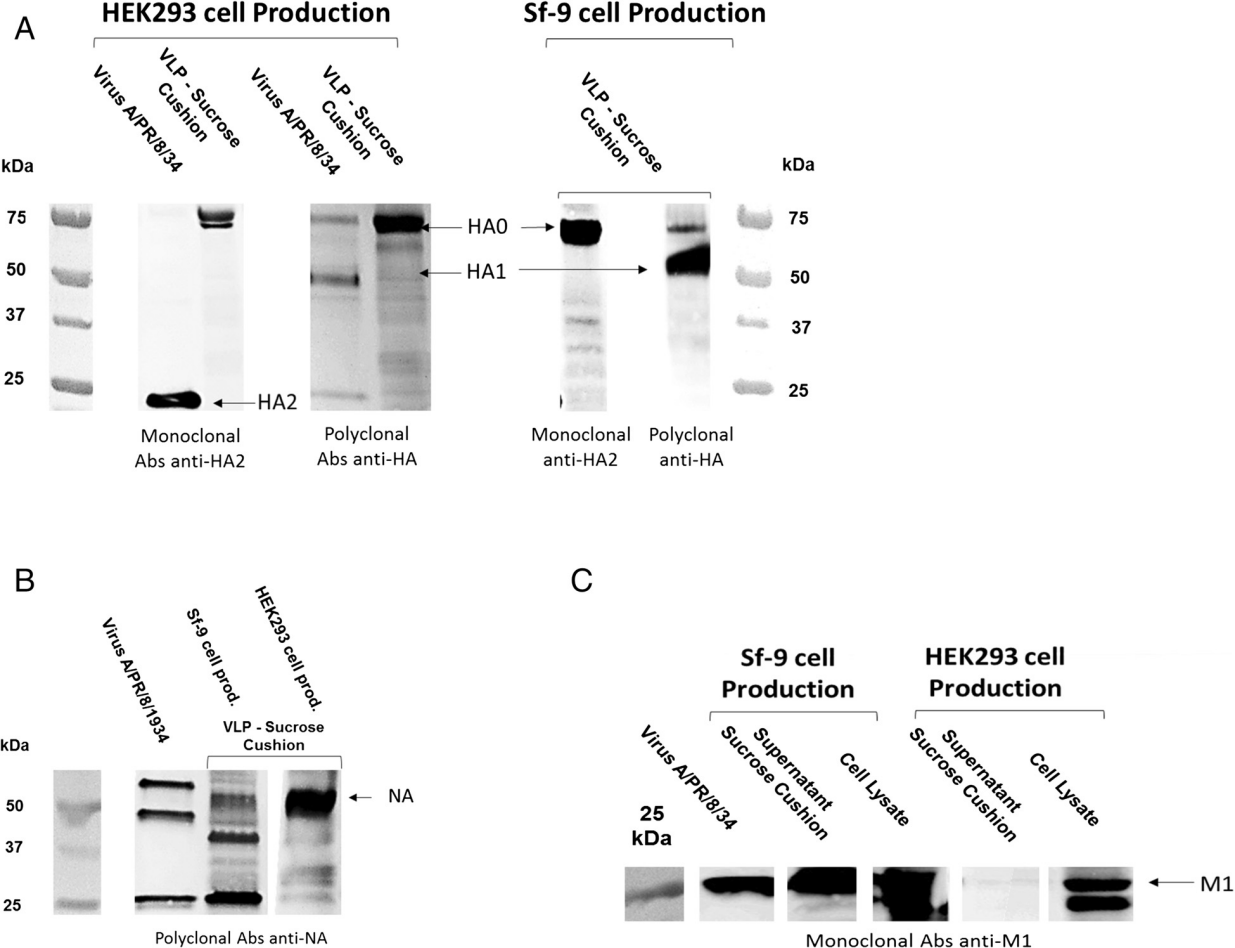
Western blot of Sucrose cushion VLP samples from HEK 293SF and Sf9 cells. **A** - Anti-HA western blot with two different antibodies, a monoclonal Abs against HA2 (Santa Cruz, sc-80550) and a polyclonal antibody against HA0 (anti-HA NIBSC sheep; 03–242). **B** - Anti-NA western blot using anti-NA NIBSC sheep (04–230). **C** - Anti-M1 western blot using monoclonal mouse antibody (Abcam, ab22396).

VLPs were expected to contain HA, NA and M1 influenza proteins. HA and NA are the antigen proteins located on the particle surface and M1 is the matrix protein that is the structural backbone of the viral particle. M1 is assumed to be located on the internal surface of the membrane envelope. For both production systems, VLP samples recovered in the culture supernatant and pre-purified by sucrose cushion were examined by Western blot. The presence of HA and NA in sucrose cushion was confirmed for VLPs produced in both the HEK293 and Sf9 cell platforms (Figure 2A & B). Surprisingly, M1 was present in the Sf9 VLPs but not found in HEK293 VLP samples (Figure 2C).

In order to confirm expression of M1 in HEK293 cells, anti-M1 Western blots of cell pellet were performed on both Sf9 and HEK293 cells (Figure 2C). M1 protein was present in both cell pellets, albeit in higher quantities in the Sf9 pellet than in HEK293. This indicated that M1 produced in HEK293 cells was not correctly incorporated into the VLPs. Integration of M1 into the Sf9 VLPs could explain for the more spherical and uniform particle shape observed with this system. M1 has been known for a long time to play an important role in determining the shape of influenza particles, especially for the formation of filamentous or spherical types of viral particles [32-34]. The M1 protein affects influenza virus morphology in a number of ways; firstly though a number of its amino acid residues [34] that interact with both HA and NA to drive the budding process [35]; and secondly, with interactions with the NP protein and the vRNP complex [36].

Studies of bud formation and viral particle morphology from the last decade proved that M1 is not the sole protein playing a role in viral particle formation and their morphology. The M2 protein, which is an ion channel protein, contains a highly conserved tail among different viral strains that was not necessarily responsible for their specific activity, but was later related to budding efficiency and particle morphology [35,37]. Besides influenza itself, host-cell heat-shock protein 70 (Hsp70) has also been described to play an important role in bud formation [38]. Actin was also reported in different studies as potentially impacting the virus produced shape [34], and a number of other cellular proteins of Vero cells have been recently detected in A/WSN/33 H1N1 influenza virions produced in this system [39]. It has also been shown for different insect cell production platforms tested (Sf9 versus Tn5 cells), that the cell type has a great impact on homogeneity of the VLPs produced [24]. Consequently, the cellular platforms used for either influenza virus or VLP production, and their membrane compositions, are expected to have a great impact on the quality of VLPs produced.

A careful review of previous influenza VLP studies indicated many contradictory results. Most of the influenza-VLP production studies consider that M1 is an important factor for correct VLP budding and is integrated into most of the constructions [19]. Chen et al. have shown that co-expression of M1 and HA proteins increases VLP production, rather than HA expressed alone [25]. But other studies have demonstrated that VLPs without M1 can be successfully produced, such as VLPs produced in HEK293T cells that only contain NA from both H5N1 and H1N1 strains [40]. Finally, M1 was also shown to form VLPs on its own in COS-1 and Sf9 cells [12,41]. This observation confirms the importance of the cellular platform on the level of incorporation of M1 in the VLPs and on particle formation, which appears to be cell line and expression system dependent.

### VLP HA activity

The particles produced as VLPs by both systems were also evaluated for their activity. The HA assay was performed to demonstrate the agglutination activity of sucrose cushion purified VLPs and the HA protein content (μg/ml) was evaluated by the standard quantification method used for influenza vaccine dose release, that is the SRID assay. Results are presented in the comparative Table 1. For the HEK293 cell production system, VLPs reached HA titers of 13 HAU/ml whereas for Sf9 cell produced VLPs, the HA activity was at 335 HAU/ml, which is more than 25 times higher. As for HA protein content, results obtained for HEK293 cells were again lower than the Sf9 cell system, with a production level of 0.092 μg HA/ml for HEK293 VLP samples and 0.38 μg HA/ml for Sf9 VLP samples (4-fold increase). It is interesting to note from a product qualification point of view that the ratio of total VLPs counted from both systems is not correlated to the results obtained from the SRID and HA assay. This is due, as discussed in a previous review of influenza-VLP quantification methods [42], to the particularity of each quantification method and their intrinsic bias. It should be emphasized that the hemagglutination or SRID assays, routinely used to quantify VLPs in past studies, do not have the ability to make a distinction between real virus-like particles and impurities [19,22]. Vesicles or baculovirus carrying HA proteins will be quantified the same way by such methods. Presently, only visual direct identification will allow for a reliable assessment of the influenza-VLP particle produced from each cellular platform. That is why this work also presents elements to qualitatively assess the purity by direct visual inspection of the samples by NSTEM in the following section.

**Table 1:** **Summary of the production performances for the two cell platform, Sf9 insect cells and HEK293SF mammalian cells**

	**Sucrose cushion samples**				**Iodixanol purified samples**		
**Cell line**	**HA protein activity (HA assay)**	**HA protein content (SRID assay)**	**VLP conc. (NSTEM)**	**Baculovirus contamination (NSTEM)**	**HA protein activity (HA assay)**	**VLP conc. (NSTEM)**	**Baculovirus contamination (NSTEM)**
**Insect Sf9**	335 HAU/ml	0.38 μg/ml	5.85 × 10^9^ VLPs/ml	1.12 × 10^11^ BV/ml	32.4 HAU/ml	2.39 × 10^9^ VLPs/ml	1.73 × 10^10^ BV/ml
**Mammalian HEK 293**	13 HAU/ml	0.092 μg/ml	1.50 × 10^8^ VLPs/ml	3.07 × 10^7^ BV/ml	8.11 HAU/ml	7.71 × 10^7^ VLPs/ml	Not quantified

Iodixanol fractions presented for both systems are the ones containing more VLP particles, i.e. fraction 3 for Sf9 cells (1.13 g/ml) and fraction 1 (1.16 g/ml) for HEK293SF cells. It should be noticed that for Sf9 and HEK293SF cells, sucrose cushions were concentrated respectively of 40 times and 50 times, and for iodixanol fractions of 110 times and 140 times. The values presented in this table represent the production levels (i.e. non concentrated samples).

### VLP purity and recovery

Zoomed-out images of sucrose cushion purified VLPs for both Sf9 cells and HEK293 cells are presented in Figure 1C & H.

#### VLP purity

For Sf9 cell productions, influenza-VLPs are identified by black arrows in Figure 1H. However, co-produced baculoviruses are also clearly identifiable in the picture (rod shaped particles, indicated by white arrows). Baculoviruses counted in this NSTEM micrograph sample were at a concentration of 1.12 × 10^11^ BV/ml, which corresponds to 20 times more than influenza-VLPs.

In order to further purify influenza-VLPs from baculoviruses, iodixanol gradient ultracentrifugation was applied and a picture of the third fraction collected (density: 1.13 g/ml) is shown on Figure 1J. Baculovirus contamination is still visible and present at a concentration of 1.73 × 10^10^ BV/ml. As a confirmation, in both sucrose cushion and further iodixanol purified samples, the presence of envelope protein GP64 from baculovirus was observed on Western blot analysis (data not shown).

Several previous studies have reported the presence of baculovirus contamination in VLP samples produced either in Sf9 or High Five cell production platforms. The level of baculovirus contamination ranged from 10^6^ to 10^8^ PFU/ml [18,22] as measured by plaque assays, which is significantly lower than the one found here. In the present work, a visual assessment of baculovirus present in the sample was preferred in order to show the total contamination present and to have a better correlation with visual VLP quantification. Baculovirus contamination in the influenza-VLP product poses a significant problem, as these viruses share structural and biophysical similarities with influenza-VLPs, thereby complicating the development of efficient and appropriate purification processes [24,26].

As previously mentioned by Tang et al., the main motivation behind producing VLPs in HEK293 cells was to avoid or reduce the baculovirus contamination [19]. In our case, and as expected, baculoviruses were present in much smaller amounts in HEK293 production samples. However, another kind of contamination was visible on HEK293 VLPs images. Cell or particle debris (dark clusters) as well as large vesicles size (>200 nm) carrying the characteristic influenza fringe can be seen. The presence of cell vesicles may also complicate purification steps in downstream processing, resulting in a similar contamination problem that exists with VLP production in the baculovirus-insect cell system (similar size of particles, vesicles carrying HA). When purified by iodixanol gradient, cell vesicles are also co-purified with lower size influenza-VLPs. Nevertheless, as indicated by the picture 1-G, high-density fractions were concentrating large cell vesicles (>300 nm).

Downstream processing and purification have now been repeatedly pointed out as a critical step and a bottleneck in the development of such new antigen design for a number of different VLPs production types [24,43]. But our observations and those of others raise additional concerns: i) the correct cut-off size for particles to be qualified as VLPs and ii) the immune response generated from co-produced vesicles as it is the case of contaminating baculoviruses.

#### VLP recovery

As we applied a concentration step by sucrose cushion ultracentrifugation prior to NSTEM analyses, in order to detect and count enough material, it was quite difficult to evaluate the proportion of VLPs recovered from each production. Although ultracentrifugation is a method broadly used to purify VLPs, it has the potential to be damaging for fragile particles. To our knowledge, VLP stability during this process has not yet been evaluated.

Nonetheless, the most likely explanation for such a high level of debris in HEK293 cell sucrose cushion samples (Figure 1D), considering that cell viability was comparable in both production systems, is that some VLPs or vesicles were disrupted by the ultracentrifugation step. The images of VLPs produced in HEK293 cells showed more debris than those of VLPs produced in Sf9 cells. This might be due to the fact that VLPs produced by HEK293 cells were more fragile than those produced in Sf9 cells. This might also be related to the observations previously made on the incorporation/lack of M1 in VLPs produced in Sf9 and HEK293 cells, suggesting the importance of M1’s presence for VLP stability. Although M1 may not be essential for VLP formation, the present study suggests its importance from a bioprocessing standpoint and the associated need for stable particles able to withstand downstream processing steps.

In order to achieve higher recovery yields, especially in the case of HEK293 cells, cell pellets from production runs were also examined. Indeed, influenza-VLPs could have been entrapped on cells due to the adhesion of HA proteins to sialic acid cellular receptors. Additionally, HEK293 cells were subjected to cell clumping, which could eventually inhibit influenza-VLPs release from the cell membrane.

**Figure 3:**
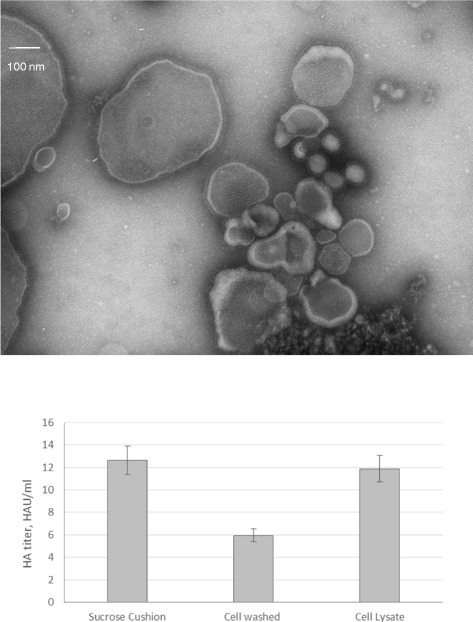
NSTEM image at 40,000× magnification of cell pellet wash upper panel and HA activity for VLP samples produced in HEK293SF cells with culture supernatant, cell pellet wash and lysate lower panel.

First, pellets of HEK293 cells were washed with a solution containing trypsin to counteract cell clumping. Second, cells were lysed. The cell pellet wash and lysate were analysed by an HA assay and by NSTEM to reveal the presence of VLPs. A treatment of the cell pellet with exogenous neuraminidase was also tested, but no noticeable effect was observed on VLP release from a pre-screening with Western blot (data not shown). A wide range of particles with a fringe were present in the sample from the cell pellet wash with trypsin on NSTEM images, including very large particles up to 500 nm (Figure 3A). A cluster of round particles approximately 100 nm in diameter could clearly be identified as influenza-VLPs. VLP concentration was counted to be 3.07 × 10^8^ VLPs/ml. Particles with a fringe were also present in the cell lysate at a concentration of 1.43 × 10^8^ VLPs/ml, and looked very similar to those presented in Figure 3A. HA assay confirmed that active HA protein was released from the cell pellet wash and in the cell lysate (Figure 3B).

Similar analyses of Sf9 cell pellet wash and cell lysate did not demonstrate comparable HA activity to the supernatant. This result was expected, as cell clumping did not occur during production. These results indicate that, for some cell production platforms, an extraction step prior to harvest should allow to increase the final VLP yields.

Influenza particles are known to bud from the cell membrane [44] and are expected to be found in the supernatant. However, other viruses, such as HIV, were reported to occasionally bud from internal multi-vesicular bodies [45], even though they usually bud from the plasma membrane. This explanation may hold some merit in our case considering that i) more than one influenza protein has the potential to induce budding [44], and ii) vesicles carrying HA and/or NA identified as VLPs may also have come from internal membranes and were released upon cell death.

### Comparison of cell platforms for VLPs production

From a basic point of view, this comparative study suggests that the Sf9 system is a better production platform. Indeed, it is more productive than the HEK293 system with 35 times more VLP particles quantified on NSTEM micrographs. The particle counts allow for comparison of cell specific productivity, as both mammalian and insect cell runs were performed at 2.0-2.5 × 10^6^ cells/ml over a course of 48 hours. Therefore, the Sf9 system also presents a significant advantage in terms of VLPs/cell productivity. Moreover, VLPs produced in insect cells were more homogeneous and resembled influenza particles produced in cell culture, whereas the VLPs produced in mammalian cells were heterogeneous in shape and size. These observations were also confirmed by HA protein quantification assays, which showed that the Sf9 cell samples had approximately 25 times more HA activity and presented higher amounts of HA concentration (μg/ml) than the HEK293 cell production system.

However, this conclusion should be nuanced by the M1 protein results and purity evaluation. If the HEK293 cell system could incorporate M1 into the influenza-VLPs, or if other matrix proteins (e.g. murine leukemia virus structural gag) would have been explored, as already done previously [46], the outcomes may have been different. In Haynes et al. [46], approximately 2×10^12^ VLP/ml were produced compared to 2×10^8^ PFU/ml of baculovirus. The increased amount of VLPs from this system compared to the present study (Table 1) could provide a better starting point when considering downstream purification of contaminates and VLP recovery for an industrial manufacturing process.

In addition to production yields and product quantification, potential safety concerns must also be considered for both production systems. Regarding cell vesicles released, it is known that they perform a variety of functions in the cell’s secretory pathway [47]. Vesicles may contain cell waste, including proteins and nucleic acids. In vaccine products, host-cell products are strongly controlled and have to be quantified and/or inactivated as indicated in regulatory agency guidelines. To our knowledge, the content of purified influenza VLPs and/or their accompanying vesicles have never been thoroughly studied. Baculovirus have also been proved to strongly initiate an immune response in mice and present adjuvanting effects when injected even at low levels with VLPs [22,48]. These effects could either benefit and boost the vaccine efficacy or create negative synergistic effects on the target VLPs immunologic response [26]. Study of baculovirus effect on mammal’s immune system is still on-going [49], and it is not presently possible to determine the safety of such contaminant in VLP products. Therefore, a lot of attention should be dedicated to the separation of VLPs from baculovirus contaminants.

## Conclusions

In this work, we have characterized VLPs using a number of established influenza assays aiming to better compare the production capabilities of two expression platforms. Such work is critical to understand and identify the bioprocessing challenges that still need to be addressed to further optimize VLP production. Moreover, the present study provides a baseline for VLP production values (Table 1) using HEK 293 and Sf9 expression systems, an information critically missing in the prevailing literature. Both systems were able to produce what is referred to as VLPs in the literature. However, this work has raised questions on what may be needed to produce structurally sound influenza VLPs, contamination issues that both systems face, and what should be defined as a virus-like particle. Influenza VLP should be a particle closely resembling influenza virus particles i.e. with the same size and morphology with the exclusion of vesicles from the cellular production system. From comparing the two production systems, it was found that the Sf9 system produced VLPs at a level 35 times more than the HEK293 system, however there remains the challenge of baculovirus contamination. Additionally, two important points should be underlined: Firstly, the incorporation of M1 in the VLP may have a significant influence on the integrity and size of VLPs produced, although further work is needed to elucidate how M1 impacts VLP stability. Secondly, the HEK293 system also presents contaminating cell vesicles, visible in a wide variety of sizes in the cell supernatant.

## Additional file

Additional file 1: Table S1. Total particle (VP/mL) and infectious particle (IVP/mL) baculovirus titres quantified by FACS and Easy titer method, respectively.

## Abbreviations

WHO: World Health Organization
VLPs: Virus-like particles
HEK293 cells: Human Embryonic Kidney 293
Sf-9 cells: Insect *Spodoptera frugiperda* cells
Tn5 cells: Insect High-Five cells
Bacmam: Baculovirus for gene transfer in mammalian cells
HA: Hemagglutinin
NA: Neuraminidase
M1: Matrix Protein 1
M2: Matrix Protein 2
NP: Nucleoprotein (NP)
vRNP: Virus ribonucleoprotein
GP64: Glycoprotein 64
BV: Baculovirus
GFP: Green fluorescent protein (GFP)
HIV: Human immunodeficient virus
SRID: Single radial immunodiffusion assay
HA assay: Hemagglutination assay
NSTEM: Negative staining transmission electron microscopy
PFU: Plaque forming units
FDA: Food and Drug Administration
GMP: Good Manufacturing Practices
CMV promotor: Cytomegalovirus promotor
polh: Polyhedron promotor
MOI: Multiplicity of infection

## References

[1] World Health Organization (WHO) (2014). Influenza (Seasonal) fact sheet. WHO media cent.

[2] Cohen J (2009). Out of Mexico? Scientists ponder swine flu’s origins. Science.

[3] Michaelis M, Doerr HW, Cinatl J (2009). Novel swine-origin influenza A virus in humans: another pandemic knocking at the door. Med Microbiol Immunol.

[4] Gerdil C (2003). The annual production cycle for influenza vaccine. Vaccine.

[5] Johnson TD (2010). Next generation of flu vaccines comming of age: cell-based technology may replace egg-based flu vaccines. Nations Health.

[6] Haynes JR (2009). Influenza virus-like particle vaccines. Expert Rev Vaccines.

[7] Kang S-M, Song J-M, Quan F-S, Compans RW (2009). Influenza vaccines based on virus-like particles. Virus Res.

[8] Pushko P, Tumpey TM, Bu F, Knell J, Robinson R, Smith G (2005). Influenza virus-like particles comprised of the HA, NA, and M1 proteins of H9N2 influenza virus induce protective immune responses in BALB/c mice. Vaccine.

[9] Wu C-Y, Yeh Y-C, Yang Y-C, Chou C, Liu M-T, Wu H-S (2010). Mammalian expression of virus-like particles for advanced mimicry of authentic influenza virus. PLoS One.

[10] Quan F-S, Vunnava A, Compans RW, Kang S-M (2010). Virus-like particle vaccine protects against 2009 H1N1 pandemic influenza virus in mice. PLoS One.

[11] Kang S-M, Yoo D-G, Lipatov AS, Song J-M, Davis CT, Quan F-S (2009). Induction of long-term protective immune responses by influenza H5N1 virus-like particles. PLoS One.

[12] Latham T, Galarza JM (2001). Formation of Wild-Type and Chimeric Influenza Virus-Like Particles following Simultaneous Expression of Only Four Structural Proteins. Journal of Virology.

[13] Pushko P, Pearce MB, Ahmad A, Tretyakova I, Smith G, Belser JA (2011). Influenza virus-like particle can accommodate multiple subtypes of hemagglutinin and protect from multiple influenza types and subtypes. Vaccine.

[14] Smith GE, Flyer DC, Raghunandan R, Liu Y, Wei Z, Wu Y (2013). Development of influenza H7N9 virus like particle (VLP) vaccine: homologous A/Anhui/1/2013 (H7N9) protection and heterologous A/chicken/Jalisco/CPA1/2012 (H7N3) cross-protection in vaccinated mice challenged with H7N9 virus. Vaccine.

[15] Landry N, Ward BJ, Trépanier S, Montomoli E, Dargis M, Lapini G (2010). Preclinical and clinical development of plant-made virus-like particle vaccine against avian H5N1 influenza. PLoS One.

[16] López-Macías C, Ferat-Osorio E, Tenorio-Calvo A, Isibasi A, Talavera J, Arteaga-Ruiz O (2011). Safety and immunogenicity of a virus-like particle pandemic influenza A (H1N1) 2009 vaccine in a blinded, randomized, placebo-controlled trial of adults in Mexico. Vaccine.

[17] D’Aoust M-A, Couture MM-J, Charland N, Trépanier S, Landry N, Ors F (2010). The production of hemagglutinin-based virus-like particles in plants: a rapid, efficient and safe response to pandemic influenza. Plant Biotechnol J.

[18] Krammer F, Schinko T, Palmberger D, Tauer C, Messner P, Grabherr R (2010). Trichoplusia ni cells (High Five) are highly efficient for the production of influenza A virus-like particles: a comparison of two insect cell lines as production platforms for influenza vaccines. Mol Biotechnol.

[19] Tang X-C, Lu H-R, Ross TM (2011). Baculovirus-produced influenza virus-like particles in mammalian cells protect mice from lethal influenza challenge. Viral Immunol.

[20] Bright RA, Carter DM, Daniluk S, Toapanta FR, Ahmad A, Gavrilov V (2007). Influenza virus-like particles elicit broader immune responses than whole virion inactivated influenza virus or recombinant hemagglutinin. Vaccine.

[21] Quan F-S, Huang C, Compans RW, Kang S-M (2007). Virus-like particle vaccine induces protective immunity against homologous and heterologous strains of influenza virus. J Virol.

[22] Margine I, Martinez-Gil L, Chou Y, Krammer F (2012). Residual baculovirus in insect cell-derived influenza virus-like particle preparations enhances immunogenicity. PLoS One.

[23] Cox MMJ, Hollister JR (2009). FluBlok, a next generation influenza vaccine manufactured in insect cells. Biologicals.

[24] Liu F, Wu X, Li L, Liu Z, Wang Z (2013). Use of baculovirus expression system for generation of virus-like particles: successes and challenges. Protein Expr Purif.

[25] Chen BJ, Leser GP, Morita E, Lamb RA (2007). Influenza virus hemagglutinin and neuraminidase, but not the matrix protein, are required for assembly and budding of plasmid-derived virus-like particles. J Virol.

[26] Vicente T, Roldão A, Peixoto C, Carrondo MJT, Alves PM (2011). Large-scale production and purification of VLP-based vaccines. J Invertebr Pathol.

[27] Côté J, Garnier A, Massie B, Kamen A, Cote J (1998). Serum-free production of recombinant proteins and adenoviral vectors by 293SF-3 F6 cells. Biotechnol Bioeng.

[28] Petiot E, Jacob D, Lanthier S, Lohr V, Ansorge S, Kamen AA (2011). Metabolic and kinetic analyses of influenza production in perfusion HEK293 cell culture. BMC Biotechnol.

[29] Alain R, Nadon F, Séguin C, Payment P, Trudel M (1987). Rapid virus subunit visualization by direct sedimentation of samples on electron microscope grids. J Virol Methods.

[30] Le Ru A, Jacob D, Transfiguracion J, Ansorge S, Henry O, Kamen AA (2010). Scalable production of influenza virus in HEK-293 cells for efficient vaccine manufacturing. Vaccine.

[31] Moulès V, Terrier O, Yver M, Riteau B, Moriscot C, Ferraris O (2011). Importance of viral genomic composition in modulating glycoprotein content on the surface of influenza virus particles. Virology.

[32] Roberts PC, Lamb RA, Compans RW (1998). The M1 and M2 proteins of influenza A virus are important determinants in filamentous particle formation. Virology.

[33] Calder LJ, Wasilewski S, Berriman JA, Rosenthal PB (2010). Structural organization of a filamentous influenza A virus. Proc Natl Acad Sci U S A.

[34] Elleman CJ, Barclay WS (2004). The M1 matrix protein controls the filamentous phenotype of influenza A virus. Virology.

[35] McCown MF, Pekosz A (2006). Distinct domains of the influenza a virus M2 protein cytoplasmic tail mediate binding to the M1 protein and facilitate infectious virus production. J Virol.

[36] Bialas KM, Bussey KA, Stone RL, Takimoto T (2014). Specific nucleoprotein residues affect influenza virus morphology. J Virol.

[37] Chen BJ, Leser GP, Jackson D, Lamb RA (2008). The influenza virus M2 protein cytoplasmic tail interacts with the M1 protein and influences virus assembly at the site of virus budding. J Virol.

[38] Watanabe K, Shimizu T, Noda S, Tsukahara F, Maru Y, Kobayashi N (2014). Nuclear export of the influenza virus ribonucleoprotein complex: Interaction of Hsc70 with viral proteins M1 and NS2. FEBS Open Bio.

[39] Shaw ML, Stone KL, Colangelo CM, Gulcicek EE, Palese P (2008). Cellular proteins in influenza virus particles. PLoS Pathog.

[40] Lai JCC, Chan WWL, Kien FF, Nicholls JM, Peiris JSM, Garcia J-M (2010). Formation of virus-like particles from human cell lines exclusively expressing influenza neuraminidase. J Gen Virol.

[41] Gómez-Puertas P, Mena I, Castillo M, Vivo A, Pérez-Pastrana E, Portela A (1999). Efficient formation of influenza virus-like particles: dependence on the expression levels of viral proteins. J Gen Virol.

[42] Thompson C, Petiot E, Lennaertz A, Henry O, Kamen A. Analytical technologies for influenza virus-like particle candidate vaccines: challenges and emerging approaches. Virology Journal. 2013;10:141; 14 pages (open access). 10.1186/1743-422X-10-141 PMC3655918 23642219

[43] Valle JR, Cha S, Medina F, Angel RM (2005). Heat shock protein 90 and heat shock protein 70 are components of dengue virus receptor complex in human cells. Society.

[44] Rossman JS, Lamb RA (2011). Influenza virus assembly and budding. Virology.

[45] Welsch S, Müller B, Kräusslich H-G (2007). More than one door - Budding of enveloped viruses through cellular membranes. FEBS Lett.

[46] Haynes JR, Dokken L, Wiley J, Cawthon AG, Bigger J, Harmsen AG (2009). Influenza-pseudotyped Gag virus-like particle vaccines provide broad protection against highly pathogenic avian influenza challenge. Vaccine.

[47] Denzer K, Kleijmeer MJ, Heijnen HF, Stoorvogel W, Geuze HJ (2000). Exosome: from internal vesicle of the multivesicular body to intercellular signaling device. J Cell Sci.

[48] Hervas-Stubbs S, Rueda P, Lopez L, Leclerc C (2007). Insect baculoviruses strongly potentiate adaptive immune responses by inducing type I IFN. J Immunol.

[49] Molinari P, Crespo MI, Gravisaco MJ, Taboga O, Morón G (2011). Baculovirus capsid display potentiates OVA cytotoxic and innate immune responses. PLoS One.

