# Supplementary material for: Critical assessment of influenza VLP production in Sf9 and HEK293 expression systems
Source: BMC Biotechnol. 2015 May 16;15:31. doi: 10.1186/s12896-015-0152-x (PMC4432999; doi:10.1186/s12896-015-0152-x)
Supplement: Additional file 1: Table S1. — Total particle (VP/mL) and infectious particle (IVP/mL) baculovirus titres quantified by FACS and Easy titer method, respectively. [file 12896_2015_152_MOESM1_ESM.doc]

Additional file 1: Table S1: Total particle (VP/mL) and infectious particle (IVP/mL) baculovirus titres quantified by FACS and Easy titer method, respectively.

| Virus Stock | BAC-HA | BAC-NA | BAC-M1 | BacMam-PR8 |
| --- | --- | --- | --- | --- |
| VP/mL | 6.46E+08 | 1.94E+09 | 3.51E+09 | 3.79E+09 |
| IVP/mL | 1.02E+08 | 1.15E+08 | 1.34E+08 | 1.21E+09 |
